# Tunable graphene micro-emitters with fast temporal response and controllable electron emission

**DOI:** 10.1038/ncomms11513

**Published:** 2016-05-10

**Authors:** Gongtao Wu, Xianlong Wei, Song Gao, Qing Chen, Lianmao Peng

**Affiliations:** 1 Key Laboratory for the Physics and Chemistry of Nanodevices, Department of Electronics, Peking University, Beijing 100871, China

## Abstract

Microfabricated electron emitters have been studied for half a century for their promising applications in vacuum electronics. However, tunable microfabricated electron emitters with fast temporal response and controllable electron emission still proves challenging. Here, we report the scaling down of thermionic emitters to the microscale using microfabrication technologies and a Joule-heated microscale graphene film as the filament. The emission current of the graphene micro-emitters exhibits a tunability of up to six orders by a modest gate voltage. A turn-on/off time of less than 1 μs is demonstrated for the graphene micro-emitters, indicating a switching speed about five orders of magnitude faster than their bulky counterparts. Importantly, emission performances of graphene micro-emitters are controllable and reproducible through engineering graphene dimensions by microfabrication technologies, which enables us to fabricate graphene micro-emitter arrays with uniform emission performances. Graphene micro-emitters offer an opportunity of realizing large-scale addressable micro-emitter arrays for vacuum electronics applications.

## Abstract (web summary)

Controlling the electron emission of microfabricated field emitters can be challenging. Here the authors report controllable and tunable graphene thermionic micro-emitters with well-defined turn-on voltages and switching times in the microsecond range and fabricate uniform micro-emitter arrays.

## Graphical abstract

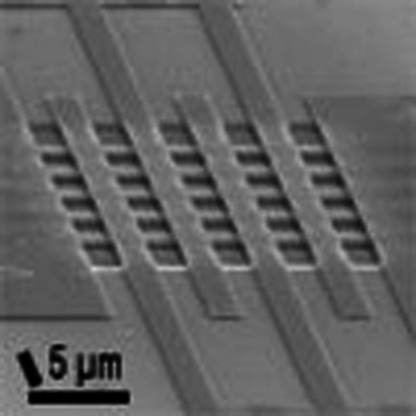

An electron emitter for generating a free electron beam is the heart of vacuum electronic devices. Owing to the simplicity of fabrication and the ease of extracting large and stable emission current, traditional thermionic electron emitters remain prevail in most commercialized vacuum electronic devices. However, because of the bulky size and slow temporal response of thermionic electron emitters, vacuum electronic devices are usually in a large volume and have a slow switching speed, which have significantly limited their applications. In 1968, Spindt pioneered the fabrication of field electron micro-emitter arrays using microfabrication technologies^1^, making it possible to scale down vacuum electronic devices to the microscale. In contrast with their bulky thermionic counterparts, field micro-emitters in an array provide near-instantaneous electron emission and their emission current can be locally tuned by an extraction gate^2^. Since then, many efforts have been made to develop microfabricated field emitters and emitter arrays^3^,^4^, including Spindt-type emitter arrays based on Mo^1^,^5^ and Si^6^,^7^ microtips and carbon nanotube emitter arrays^8^,^9^, and many novel applications in vacuum electronics emerged, for example, microscale vacuum transistors^10^,^11^,^12^, flat panel displays^13^,^14^, X-ray tubes for dynamic X-ray detection^15^ and so on. However, because of the highly sensitive dependence of field emission on the atomic structures of emitter tips and the difficulty in controlling them^3^, controllable electron emission from microfabricated field emitters still proves challenging. As a result, emission current corresponding to a fixed extraction voltage usually varies from emitters to emitters in an emitter array, which seriously degrades its performances and hinders its practical applications. As electrons are believed to be mainly emitted from the atoms locating at the topmost tip in field emission^16^ and the precise engineering of the tip structures at the atomic scale by traditional microfabrication technologies is considered to be extremely difficult, if not impossible, controllable field emission from micro-emitter tips seems to be an insurmountable challenge. To this end, scaling down thermionic electron emitters to the microscale by microfabrication technologies may provide an alternative route to achieve microscale emitters and emitter arrays for practical applications in vacuum electronics, considering their insensitivity to the atomic structures of emitters and the great successes of thermionic emitters already in vacuum electronics. Moreover, as a result of the decrease of thermal inertia, the temporal response of the miniaturized thermionic emitters is expected to be significantly speeded up as compared with their bulky counterparts and may be not a limiting factor for the applications of thermionic emitters any more. However, to date, no such attempt has been reported to scale down thermionic emitters to the microscale and construct thermionic micro-emitter arrays by microfabrication technologies.

Regarding the materials for the filaments of microfabricated thermionic emitters, it is highly desired from them to exhibit good electrical conductivity, high decomposition or melting temperature, proper work function and good chemical inertness, as learnt from the properties of the filaments of bulky thermionic emitters. More importantly, they have to be compatible with traditional microfabrication technologies and can be controllably tailored into microscale dimensions by microfabrication technologies. In recent years, two-dimensional layered materials have attracted great research interests because of their promise in field effect transistors^17^,^18^, photoelectric devices^19^,^20^, field electron emitters^21^,^22^,^23^,^24^ and so on, and many of those devices are fabricated by microfabrication technologies because of their compatible fabrication scheme with the latter. Among all known two-dimensional materials, graphene acts as the ideal candidate for the filament of microfabricated thermionic emitters and emitter arrays as it meets all above requirements for the filaments of microfabricated thermionic emitters^17^,^25^,^26^. Moreover, graphene has been studied to exhibit significant thermionic emission current^27^,^28^.

In this paper, we report the miniaturization of thermionic emitters and the construction of the micro-emitter arrays by using microfabrication technologies and employing Joule-heated microscale graphene films as the filaments. Emission current of the graphene micro-emitters (GMEs) measured by a top electrode can be tuned by up to six orders of magnitude by a modest gate voltage of approximately 15 V, and a turn-on/off time of less than 1 μs is demonstrated for a GME. In contrast with microfabricated field emitters, whose turn-on voltage is difficult to control through microfabrication, the turn-on voltage of GMEs depends on graphene dimensions, enabling controllable and reproducible emission performances of GMEs by engineering graphene dimensions by microfabrication technologies. Consequently, micro-emitter arrays consisting of GMEs with uniform electron emission performances are successfully realized. The large-magnitude tunability by a modest gate voltage, fast temporal response and good controllability of emission performances make GMEs promising in realizing large-scale addressable micro-emitter arrays for vacuum electronics applications.

## Results

### Structure of a GME and its tunable emission current

**Figure 1:**
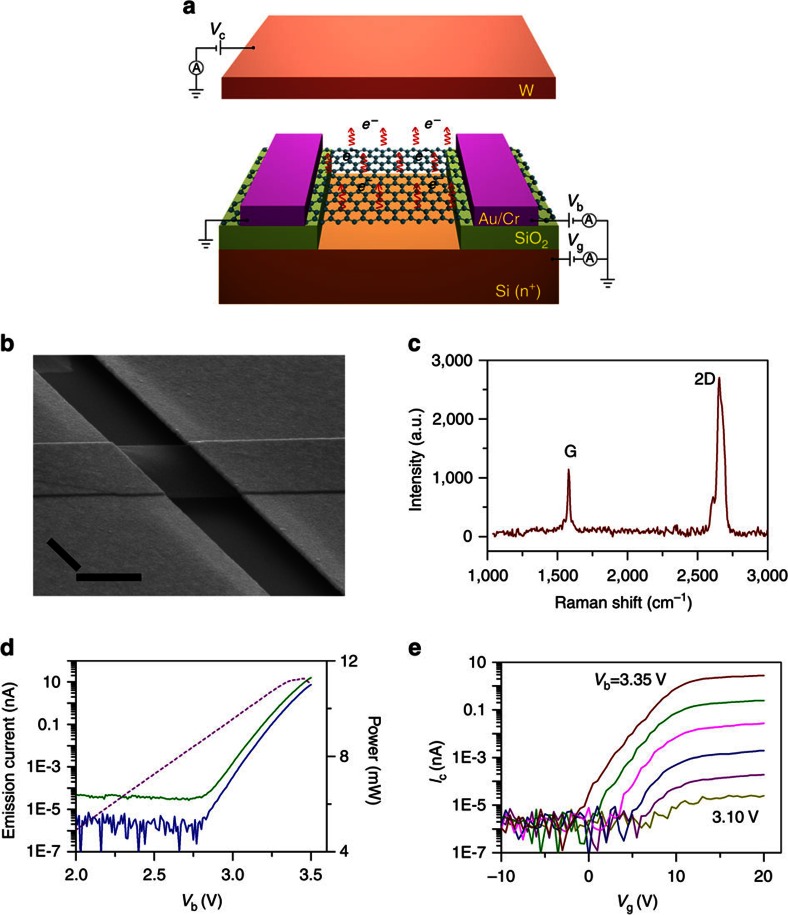
Structure and electron emission performances of a GME. (**a**) A schematic drawing showing the structure of a GME and its working principle. A GME is fabricated on a SiO_2_/Si wafer substrate and consists of a graphene film suspended between two metal (Au/Cr) electrodes and over the bottom Si electrode. A bias voltage (*V*_b_) is applied to a GME to drive the electron emission from it and a top W electrode with a collecting voltage (*V*_c_) applied is used to collect and measure the emission current. The highly doped Si layer applied with a voltage of *V*_g_ acts as the gate to tune the emission current measured by the top W electrode. (**b**) Tilted SEM image of a GME (scale bar, 1 μm). (**c**) Raman spectrum of a GME exhibiting a G peak of 1,580 cm^−1^ and a 2D peak of 2,656 cm^−1^. (**d**) Emission current of a GME measured by the upper collector electrode (*I*_c_, solid blue line) and the bottom gate electrode (*I*_g_, solid olive line) and the corresponding applied Joule-heating power (dashed purple line) when ramping up *V*_b_ and fixing *V*_c_ (100 V) and *V*_g_ (15 V). (**e**) *I*_c_−*V*_g_ curves of the same GME as in **c** when *V*_b_ increases from 3.10 to 3.35 V at 0.05 V intervals and *V*_c_ is fixed at 100 V.

Figure 1a schematically shows the structure of a GME fabricated on a SiO_2_/Si wafer by microfabrication technologies. It consists of a microscale graphene film suspended between two Au/Cr electrodes and over a heavily doped Si bottom electrode. A scanning electron microscope (SEM) image of a GME is shown in Fig. 1b. Figure 1c is the Raman spectrum of a GME, indicating a layer number of 1 or 2 for the graphene emitter^29^,^30^. To drive electron emission from a GME, a bias voltage (*V*_b_) and thus an electric current (*I*_b_) are applied to the graphene emitter through the two Au/Cr electrodes to heat it up by Joule heating. To measure the emission current, a collecting voltage (*V*_c_) is applied to a W electrode locating ∼200 μm above the graphene emitter. The heavily doped Si bottom electrode applied with a gate voltage (*V*_g_) works as a gate electrode to tune the emission current measured by the upper W electrode. Figure 1d shows the emission current (*I*_c_) from a graphene emitter measured by the upper W electrode together with the current (*I*_g_) measured by the bottom gate when *V*_b_ was ramped up from 2.0 to 3.5 V and *V*_c_ and *V*_g_ were fixed at 100 and 15 V, respectively. It can be seen that electron emission from the GME takes place at a turn-on voltage of only ∼2.8 V. When *V*_b_ is larger than the turn-on voltage, both *I*_c_ and *I*_g_ increase exponentially with *V*_b_. The two currents increase synchronously with *I*_g_ being about two times larger than *I*_c_, which is attributed to the larger collecting electric field (∼50 V μm^−1^) between graphene and the bottom gate than that between graphene and the top W electrode (∼0.5 V μm^−1^). Further experimental evidences to indicate that the measured electron emission is from graphene film are shown in Supplementary Fig. 1.

Emission current of a GME measured by the upper W electrode is observed to be tuned by a large magnitude through a modest voltage applied to the bottom gate. Figure 1e shows the measured *I*_c_–*V*_g_ curves of the same GME as that in Fig. 1d when *V*_b_ increases from 3.10 to 3.35 V at 0.05 V intervals and *V*_c_ is fixed at 100 V. A *V*_c_ value of 100 V ensures that all those curves were measured at accelerating field regime without the retardation of space charge (see Supplementary Fig. 2 for details). It can be seen that, in contrast with the weak tunability of the graphene conductivity (see Supplementary Fig. 3 for details), *I*_c_ exhibits a tunability of large magnitude and high efficiency by *V*_g_. At *V*_b_=3.35 V, *I*_c_ is tuned by a magnitude of up to six orders when *V*_g_ increases from −1.5 to 13 V, corresponding to an average *V*_g_ of only ∼2.4 V needed to tune *I*_c_ by ten times. This is much more efficient than that of field micro-emitters, which usually needs a voltage of at least more than 10 V to achieve the same magnitude tunability^4^,^31^. For each *I*_c_–*V*_g_ curves, there are three regimes with the increase of *V*_g_. In the first regime, there is no measurable emission current, indicating that emission current measured by the top W electrode is completely suppressed by the bottom gate. In the second regime, emission current becomes measureable and increases approximately exponentially with *V*_g_. In the third regime, emission current increases slowly with *V*_g_ and approaches saturation. The special property of the GME enables us to switch on/off a specific GME in an array completely and efficiently by a local bottom gate, making it addressable by a *V*_g_. With the increase of *V*_b_ at fixed intervals of 0.05 V, the threshold gate voltage for turning on the GME decreases at approximately constant intervals of ∼2.0 V, whereas emission current corresponding to different *V*_b_ saturates invariably at a *V*_g_ of around 12 V (Fig. 1e).

**Figure 2:**
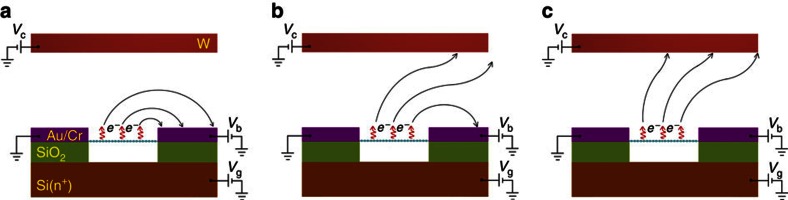
Schematic mechanism of gate tunability. (**a**) All electrons emitted from a GME are collected by a side electrode in the first regime of the *I*_c_−*V*_g_ curves in Fig. 1e, where *I*_c_ is completely suppressed by *V*_g_. Solid arrowed lines indicate electron trajectories. (**b**) Part of electrons are collected by the top electrode in the second regime of the *I*_c_−*V*_g_ curves where *I*_c_ increases fast with *V*_g_. (**c**) All electrons are collected by the top electrode in the third regime of the *I*_c_−*V*_g_ curves, where *I*_c_ approaches saturation.

As the temperature of a graphene emitter under a fixed bias voltage was found to exhibit a minor increase with the increase of *V*_g_ (see Supplementary Fig. 4 for details) and the decrease of work function due to electrostatic doping by *V*_g_ is estimated to be negligible (see Supplementary Discussion for details), the above large magnitude tunability of *I*_c_ cannot be attributed to tunable emission current density of graphene itself. To figure out the mechanism responsible for the large magnitude tunability of *I*_c_ by *V*_g_, we simulated electron trajectories in our measurement setup (see Supplementary Fig. 5 for details). It can be seen from Supplementary Fig. 5b that electrons emitted from the graphene film are all projected to the side Au/Cr electrode with positive *V*_b_ applied when *V*_g_=−10 V (Fig. 2a), which explains well why *I*_c_ is completely suppressed in the first regime. In the second regime, electrons start to be collected by the top W electrode (see Fig. 2b and Supplementary Fig. 5c for details), and more and more electrons are collected by the top W electrode with the increase of *V*_g_ until almost all electrons are collected by the top W electrode in the third regime (see Fig. 2c and Supplementary Fig. 5d). Therefore, *I*_c_ increases fast with *V*_g_ in the second regime and approaches saturation in the third one. Resembling to the gate tunability of traditional vacuum triodes, the large magnitude tunability of *I*_c_ by *V*_g_ is attributed to the steering of electron trajectories by the gate electrode, which controls the ratio of electrons arriving at the collector electrode to all those emitted from graphene. The mechanism is schematically shown in Fig. 2.

### Temporal response of a GME

**Figure 3:**
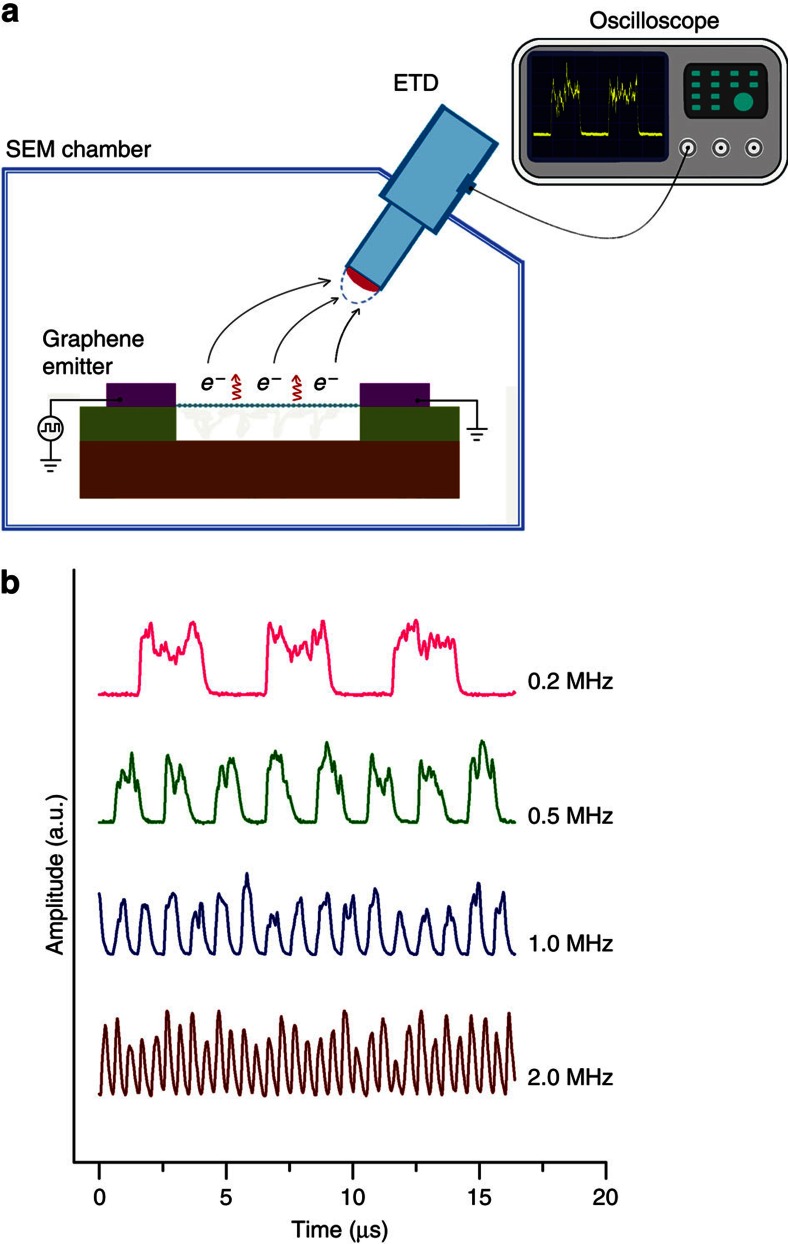
Temporal response of a GME. (**a**) A schematic drawing showing the measurement of the temporal response of a GME inside a SEM chamber. Electron emission from a GME is driven by a square wave voltage with the high and low voltage levels larger and smaller than the turn-on voltage of the GME, respectively. Electron emission is detected by the ETD of a SEM and recorded by an oscilloscope. (**b**) Output signals of ETD when electron emission from a GME is driven by a square wave voltage of different frequencies.

Temporal response of an electron emitter is an important parameter for measuring the maximum rate of switching it on/off and fast temporal response is highly desired for the applications of electron emitters in the area requiring a fast alternating electron beam. To accurately measure the temporal response of a GME, which has a relatively small emission current in the order of nA, we use the Everhart–Thornley detector (ETD) of a SEM to detect the electrons emitted from a GME (Fig. 3a). Electron emission from an emitter was driven by a square wave voltage with a low and high voltage level of 2.23 and 2.63 V, respectively, and a duty ratio of 50%. The low and high voltage levels were selected to be smaller and larger than the turn-on threshold voltage of the GME (∼2.40 V), so electron emission is expected to take place only in the period of high voltage level if the temporal response of the GME is fast enough. Figure 3b shows the output signals of ETD corresponding to the input square wave voltage of different frequencies (see Supplementary Fig. 6 for experimental evidences to indicate that the ETD signal originates from electron emission from a graphene film). It can be seen that a fast response frequency of up to 2 MHz can be achieved for the GME, indicating a turn-on/off time of less than 1 μs. Owing to the impedance of the input circuit (see Supplementary Fig. 7 for details), the intrinsic turn-on/off time of the GME is thought to be much smaller than the value obtained here. The fast temporal response of electron emission from a GME is attributed to the fast response of its temperature change resulted from the scaling down of the filament dimensions, as the rate of turning-on/off electron emission from a Joule-heated graphene is limited by the process of building a steady temperature distribution under Joule-heating^32^,^33^. Theoretically, a GME can reach a steady temperature in a time ∼20 ns when it is heated by Joule-heating and can be cooled down in 1.3 ns when turning it off (see Supplementary Fig. 8 for details).

### Electron emission of GMEs with different graphene dimension**s**

**Figure 4:**
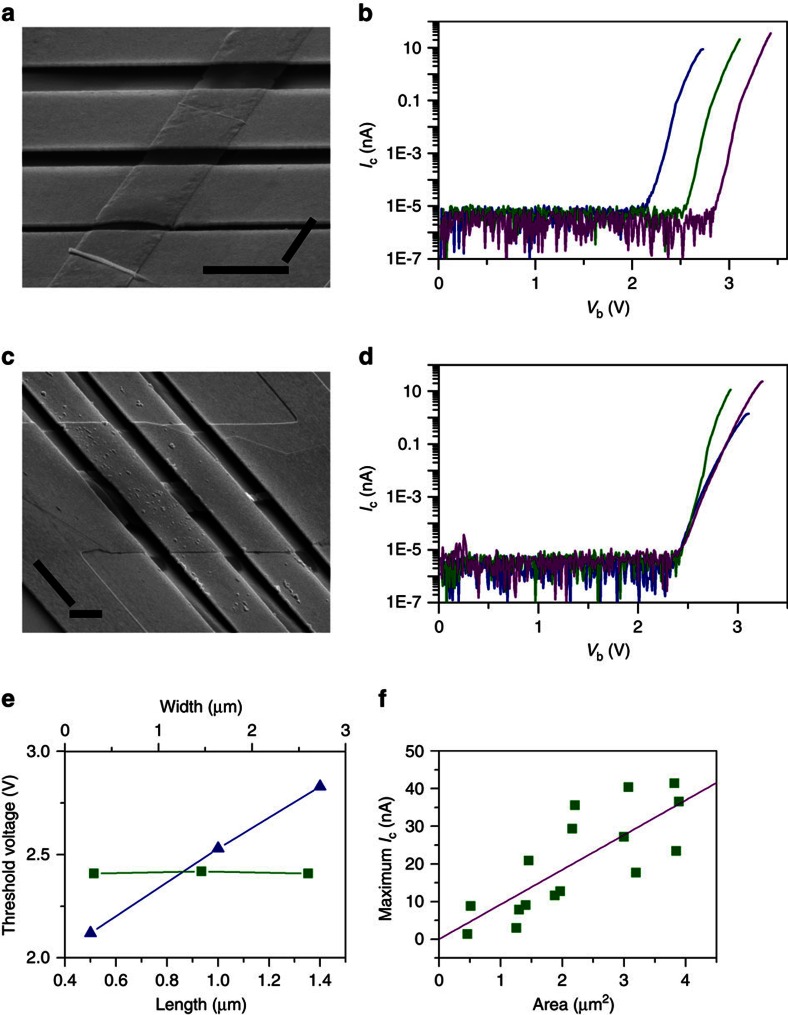
Electron emission performances of GMEs with different graphene dimensions. (**a**) Tilted SEM image of three GMEs with the same width of 1.7 μm but different length of 0.5, 1.0 and 1.4 μm, respectively, after breakdown (scale bar, 2 μm). (**b**) *I*_c_−*V*_b_ curves of the GMEs with the length of 0.5 μm (blue line), 1.0 μm (olive line) and 1.4 μm (purple line) as shown in **a**. (**c**) Tilted SEM image of three GMEs with the same length of 1.5 μm but different widths of 0.3, 1.5 and 2.6 μm, respectively, after breakdown (scale bar, 2 μm). (**d**) *I*_c_–*V*_b_ curves of the GMEs with the width of 0.3 μm (blue line), 1.5 μm (olive line) and 2.6 μm (purple line) as shown in **c**. *I*_c_−*V*_b_ curves in **b** and **d** were measured until the breakdown of GMEs due to excess electrical and thermal stress. (**e**) The plots of the turn-on threshold bias voltage versus the length (blue triangles) and width (olive squares) of the GMEs in **b** and **d**. (**f**) Maximum emission current (olive squares) of GMEs with different area. The solid line is the linear fitting of the data points. All data shown in this figure were measured at *V*_c_=100 and *V*_g_=10 V.

The threshold bias voltage for turning on a GME is observed to exhibit definite relationships with the dimensions of graphene emitters. Figure 4 shows the electron emission performances of GMEs with different graphene dimensions. For the GMEs with the same width of 1.7 μm but different length of 0.5, 1.0 and 1.4 μm (Fig. 4a), they have a turn-on voltage of 2.1, 2.5 and 2.8 V, respectively, exhibiting an approximately linear dependence on the length of GMEs (Fig. 4b,e). For the GMEs with the same length of 1.5 μm, but different width of 0.3, 1.5 and 2.6 μm (Fig. 4c), they have the same threshold voltages of around 2.4 V (Fig. 4d,e), indicating that the turn-on voltage of GMEs exhibits no obvious dependence on the width of graphene emitters. The definite dependences of turn-on voltage on graphene dimensions enable the well control of turn-on voltage through engineering graphene dimensions, which can be easily achieved by traditional microfabrication technologies^17^. Moreover, as shown in Fig. 4b,d, GMEs with different length or width have similar increasing slope of *I*_c_ with *V*_b_ after being turned on (except the GME with a width of 1.5 μm in Fig. 4d, probably due to its structural defects), so the successful control of the turn-on voltage will result in the successful control of the overall electron emission performances of GMEs.

The *I*_c_–*V*_b_ curves in Fig. 4b,d were measured by ramping up *V*_b_ until GMEs broke down due to the excess thermal and electrical stress. The maximum emission current of GMEs before breakdown is observed to increase with the length (Fig. 4b) and the width (Fig. 4d) of graphene emitters. Figure 4f plots the maximum emission current of 16 graphene emitters versus their area. It can be seen that the maximum emission current of graphene emitters increases approximately linearly with their area, indicating that electrons are emitted mainly through graphene surface but not the edges. A linear fitting to the plot in Fig. 4f gives an averaged maximum emission density of 0.92 A cm^−2^. The maximum emission current density of graphene emitters exhibits no obvious dependence on their thickness and a thick graphene emitter with a layer number of ∼52 was found to exhibit similar emission density to those of one or two layer graphene (see Supplementary Fig. 9 for details). SEM images of graphene emitters in Fig. 4a,c show that the graphene emitters broke down along their middle line without obvious narrowing. This agrees well with the simulated temperature distribution of a graphene emitter that reaches maximum along its middle line (Supplementary Fig. 8c). A graphene emitter is expected to exhibit the highest emission current density near the hottest site along its middle line^33^.

## Discussion

GMEs possess several advantages in fabrication and performances when compared with traditional thermionic electron emitters. First, in contrast with the fabrication of bulky thermionic emitters using traditional machining methods, GMEs are fabricated using microfabrication technologies. In addition to the great increase of fabrication efficiency, this will make it possible to combine GME-based vacuum electronic devices and solid-state ones to achieve some new functions by integrating them on the same wafer substrate. Moreover, microfabrication technologies, together with the successful growth of high-quality wafer-scale graphene samples^34^, enable the fabrication and integration of large-area GME arrays for large-scale applications. Second, as shown in Fig. 1e, emission current of a GME can be locally tuned by a large magnitude through a modest gate voltage, which is highly desired for constructing addressable large-scale emitter arrays for the applications in vacuum microelectronics. Third, the temporal response of GMEs (less than 1 μs as shown in Fig. 3b) is about five orders of magnitude faster than that of its traditional bulky counterparts, usually hundreds of milliseconds. The fast temporal response of GMEs make them promising in the applications requiring a fast switching of electron beam, like X-ray tubes for dynamic X-ray detections^15^. Although vacuum electronics devices are usually in a large volume and have a slow switching speed because of the bulky size and slow temporal response of traditional thermionic emitters, the microscale size of GMEs and its fast temporal response provide a promising way of scaling down vacuum electronic devices to the microscale and speeding them up. Therefore, the scaling down of thermionic emitters to the microscale using microfabrication technologies and employing graphene films as the filaments endows them with many new opportunities in vacuum electronics applications.

**Figure 5:**
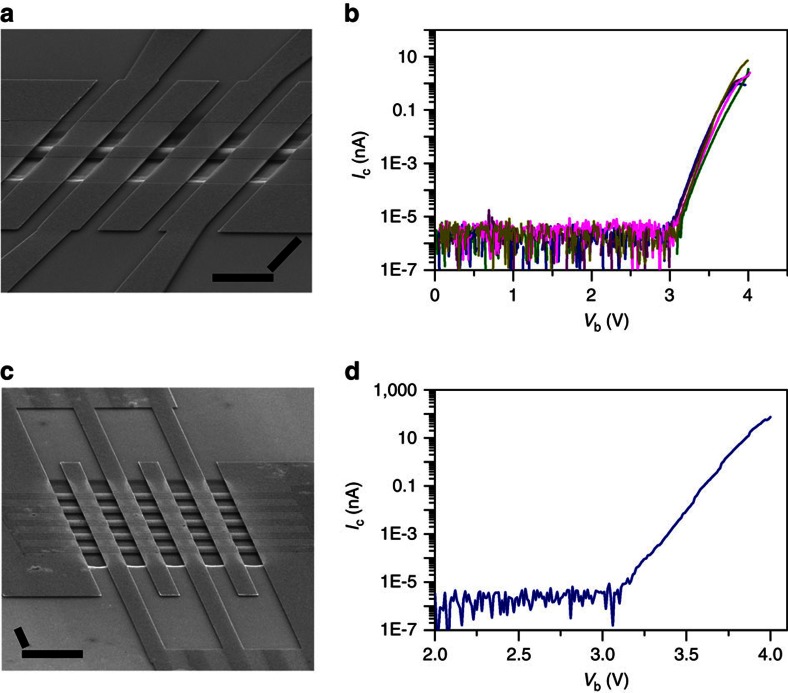
Electron emission performances of GME arrays. (**a**) Tilted SEM image of a row of five GMEs having same dimensions. (**b**) *I*_c_−*V*_b_ curves of individual GMEs in the array shown in **a**. They are displayed in sequence in blue, olive, purple, magenta and dark yellow, respectively, corresponding to the GMEs from left to right in **a**. (**c**) Tilted SEM image of a 5 × 5 parallel GME array connected by interdigital electrodes with each GME having same dimensions as those in **a**. (**d**) *I*_c_−*V*_b_ curve of the array in **c**. *I*_c_–*V*_b_ curves in **b** and **d** were measured at *V*_c_=100 V and *V*_g_=10 V. The scale bars in **a** and **c** are 5 μm.

Even though field micro-emitters and micro-emitter arrays have already been fabricated using microfabrication technologies for decades and possesses the same merits of being integratable, fast temporal response and local gate tunability as GMEs, controllable electron emission from microfabricated field micro-emitters still proves challenging^3^,^35^, making it difficult to fabricate field micro-emitters with desired emission performances in a reproducible manner. Compared with uncontrollable electron emission from field micro-emitters, the definite dependences of the emission performances of GMEs on graphene dimensions in Fig. 4 enable us to fabricate GMEs with controllable and reproducible emission performances through graphene dimension engineering. Figure 5a shows a row of five GMEs with the same dimensions of 1.6 × 1.8 μm^2^. It can be seen from Fig. 5b that the five GMEs have quite reproducible electron emission performances with nearly the same turn-on bias voltages of around 3.10 V (3.05, 3.06, 3.10, 3.13 and 3.15 V, respectively) with a spread of 0.1 V and quite similar increasing rates of emission current with the increase of bias voltage. The reproducible emission performances of GMEs allow us to fabricate a parallel GME array with all GMEs in the array having uniform emission performances if they are engineered into the same dimensions, which ensures that all GMEs in the array can be switched on/off synchronously and have quite similar emission current at a fixed working voltage.

Figure 5c shows a 5 × 5 GME array with all the GMEs connected in parallel to two interdigital electrodes and having the same dimensions as those of the GMEs in Fig. 5a. The electron emission performance in Fig. 5d indicates that the 5 × 5 GME array has the same turn-on voltage of 3.10 V as that of individual emitters shown in Fig. 5b. This makes it feasible to control the turn-on voltage of a parallel GME array by controlling the turn-on voltage of individual GMEs in the array through engineering graphene dimensions. To evaluate the uniformity of the GMEs in the parallel array, we compare the emission current of the array to that of individual emitters in Fig. 5b considering they have the same turn-on voltages. At *V*_b_=4.0 V, the GME array has an emission current of 74.5 nA, whereas the individual GMEs in Fig. 5b have an average emission current of 3.0 nA. The former is about 25 times larger than the latter, in good agreement with the number of the emitters in the array. Therefore, the emitters in the array have good uniformity and contribute nearly equally to the emission current of the array. A single GME could be used to light up a fluorescent screen (see Supplementary Fig. 10 for details), which shows great promise of GME arrays for the applications in flat panel displays.

In addition to the controllable and reproducible emission performances of GMEs, electron emission from a GME and a GME array can be driven by a voltage of as low as ∼2–3 V depending on graphene length (Figs 4 and 5). This is much lower than the working voltage of field micro-emitters, which usually needs at least tens of volts to initiate the field emission^3^,^4^. More importantly, benefit from the thermionic emission scheme GMEs adopt and the chemical inertness of graphene, GMEs exhibit stable electron emission at a relatively poor vacuum conditions of ∼10^−3^ Pa (see Supplementary Fig. 11 for details), which is much lower than that needed for stable field emission (<10^−6^ Pa). The low vacuum requirement for the operation of GMEs will significantly decrease the cost of GME-based vacuum electronic devices.

Emission current of a GME is observed to exhibit a strong correlation with the bias voltage or internal electric field applied to it, rather than the applied Joule-heating power (Fig. 1d). An increase of emission current with the decrease of Joule-heating power is even observed when *V*_b_ is larger than 3.4 V in Fig. 1d. This indicates that the mechanism responsible for the electron emission from a GME should be different from that for a bulky thermionic emitter, where electron emission is driven by the thermal effect and emission current of a given emitter increases with the emitter temperature and thus applied Joule-heating power. Our previous studies have shown that because of the accumulation of electric-field-induced hot electrons, electron emission from a Joule-heated carbon nanotube and graphene nanoribbon can be directly and mainly driven by internal electric field in addition to the thermal effect^32^,^33^,^36^. The accumulation of electric-field-induced hot electrons in suspended Joule-heated graphene films was recently reported to explain the enhanced thermal light emission from them^37^. Our measured electron emission from GMEs is thus thought to be mainly driven by internal electric field as well due to the accumulation of electric-field-induced hot electrons. This explains well why emission current of a GME exhibits a strong correlation with internal electric field applied to it, rather than the applied Joule-heating power. It is worth noting that even though the accumulation of electric-field-induced hot electrons in a Joule-heated filament is expected to enhance its emission current density^33^,^36^, the exceptional performances of a GME, for example, large-magnitude tunability, fast temporal response, good controllability and so on, are not related to it.

To summarize, thermionic emitters are scaled down to the microscale for the first time by using microfabrication technologies and employing microscale Joule-heated graphene films as the filaments. Compared with traditional bulky thermionic emitters, GMEs possess the merits of microscale size, fast temporal response, large-magnitude local gate tunability by a modest gate voltage and being integratable by microfabrication technologies. Compared with microfabricated field emitters, GMEs exhibit controllable and reproducible electron emission performances, low working voltage and low requirement for operation vacuum. Possessing the advantages of both bulky thermionic emitters and microfabricated field emitters, GMEs open up a route of realizing large-scale addressable micro-emitter arrays for vacuum electronics applications. Because of the relative high work function of graphene and the high dissipation rate of Joule-heating power, emission efficiency and emission current density of GMEs are still overshadowed compared with those of microfabricated field emitters and have room for improvement for practical applications. In the future, they may be improved by lowering the work function of graphene by chemical/electrostatic doping or employing other low-dimensional materials with lower work functions as the filaments.

## Methods

### GME microfabrication

GMEs and GME arrays are fabricated on SiO_2_/Si wafer substrates with a SiO_2_ layer thickness of 300 nm using microfabrication technologies. Graphene films are first obtained by mechanical exfoliation of a thin foil of highly oriented pyrolytic graphite and the films with a thickness of one to three layers as determined from their contrast under optical microscope observation are selected for GME fabrications. The selected graphene films are first tailored to ribbons in pre-designed dimensions and configurations by electron beam lithography (EBL) followed by plasma etching, then metal electrodes (70 nm Au/5 nm Cr) are fabricated using standard EBL, metal film deposition and lift-off processes. To make graphene ribbons suspended, the SiO_2_ layer underneath them is removed by chemical etching. The SiO_2_/Si wafer is first coated by a layer of polymethyl methacrylate (PMMA) film acting as the mask and small windows in the PMMA mask are opened by EBL to expose the areas for chemical etching, then the wafer is immersed in buffered hydrofluoric acid to remove SiO_2_ layer. After removing the PMMA mask and drying the wafer in hot isopropanol, graphene emitters suspended between two metal electrodes and over the bottom highly doped Si electrode are obtained. All GMEs in an array are fabricated from the same original graphene films with uniform thickness.

### Electron emission performance measurement

Electron emission performances of GMEs in direct current mode are measured on a probe station (Lakeshore TTP4) by using a Keithley 4200 semiconductor characterization system. A W probe is used as the top collector electrode. The temporal response of GMEs is studied inside a FEI Quanta 600 F SEM by using a Kleindiek MM3A probing system to achieve electrical connections. Square wave signal is input by a waveform generator (Agilent 33220A) and the output signal of ETD is detected and recorded by an oscilloscope (Aglient DSO7054A). When measuring the temporal response of GMEs, the electron beam of SEM is blanked, and the grid voltage and scintilator voltage of ETD are set to be 210 and 10,000 V, respectively. All measurements are performed at room temperature and a vacuum level of ∼10^−3^ Pa.

## Additional information

**How to cite this article:** Wu, G. *et al*. Tunable graphene micro-emitters with fast temporal response and controllable electron emission. *Nat. Commun.* 7:11513 doi: 10.1038/ncomms11513 (2016).

## Supplementary Material

Supplementary Information Supplementary Figures 1-11, Supplementary Discussion and Supplementary References

## References

[b1] Spindt C. A. A thin-film field-emission cathode. J. Appl. Phys. 39, 3504–3505 (1968).

[b2] Chubun N. N. . Fabrication and characterization of singly addressable arrays of polysilicon field-emission cathodes. Solid State Electron 45, 1003–1007 (2001).

[b3] Xu N. S. & Huq S. E. Novel cold cathode materials and applications. Mater. Sci. Eng. R 48, 47–189 (2005).

[b4] Temple D. Recent progress in field emitter array development for high performance applications. Mater. Sci. Eng. R 24, 185–239 (1999).

[b5] Spindt C. A. . Physical properties of thin-film field emission cathodes with molybdenum cones. J. Appl. Phys. 47, 5248–5263 (1976).

[b6] Huq S. E., Chen L. & Prewett P. D. Sub10nm silicon field emitters produced by electron beam lithography and isotropic plasma etching. Microelectron. Eng. 27, 95–98 (1995).

[b7] She J. C. . Silicon tip arrays with ultrathin amorphous diamond apexes. Appl. Phys. Lett. 81, 4257–4259 (2002).

[b8] Teo K. B. K. . Carbon nanotubes as cold cathodes. Nature 437, 968–968 (2005). 16222290 10.1038/437968a

[b9] Wu J. F. . Fabrication and field emission properties of triode-type carbon nanotube emitter arrays. Nano Lett. 9, 595–600 (2009). 19161333 10.1021/nl802777g

[b10] Pescini L. . Nanoscale lateral field-emission triode operating at atmospheric pressure. Adv. Mater. 13, 1780–1783 (2001).

[b11] Han J.-W., Oh J. S. & Meyyappan M. Vacuum nanoelectronics: back to the future?-Gate insulated nanoscale vacuum channel transistor. Appl. Phys. Lett. 100, 213505 (2012).

[b12] Srisonphan S., Jung Y. S. & Kim H. K. Metal-oxide-semiconductor field-effect transistor with a vacuum channel. Nat. Nanotechnol 7, 504–508 (2012). 22751220 10.1038/nnano.2012.107

[b13] Choi W. B. . Fully sealed, high-brightness carbon-nanotube field-emission display. Appl. Phys. Lett. 75, 3129–3131 (1999).

[b14] Liu P. . New-type planar field emission display with superaligned carbon nanotube yarn emitter. Nano Lett. 12, 2391–2396 (2012). 22494219 10.1021/nl3003792

[b15] Parmee R. J. . X-ray generation using carbon nanotubes. Nano Convergence 1, 1–27 (2014).

[b16] Zhang H. . Nanostructured LaB_6_ field emitter with lowest apical work function. Nano Lett. 10, 3539–3544 (2010). 20715844 10.1021/nl101752z

[b17] Novoselov K. S. . Electric field effect in atomically thin carbon films. Science 306, 666–669 (2004). 15499015 10.1126/science.1102896

[b18] Radisavljevic B. . Single-layer MoS_2_ transistors. Nat. Nanotechnol 6, 147–150 (2011). 21278752 10.1038/nnano.2010.279

[b19] Echtermeyer T. J. . Strong plasmonic enhancement of photovoltage in graphene. Nat. Commun. 2, 458 (2011). 21878912 10.1038/ncomms1464

[b20] Lopez-Sanchez O. . Ultrasensitive photodetectors based on monolayer MoS_2_. Nat. Nanotechnol 8, 497–501 (2013). 23748194 10.1038/nnano.2013.100

[b21] Rout C. S. . Superior field emission properties of layered WS_2_-RGO Nanocomposites. Sci. Rep. 3, 3282 (2013). 24257504 10.1038/srep03282 PMC3836036

[b22] Rout C. S. . Enhanced field emission properties of doped graphene nanosheets with layered SnS_2_. Appl. Phys. Lett. 105, 043109 (2014).

[b23] Late D. J. . Pulsed laser-deposited MoS_2_ thin films on W and Si: field emission and photoresponse studies. ACS Appl. Mater. Interfaces 6, 15881–15888 (2014). 25141299 10.1021/am503464h

[b24] Kashid R. V. . Enhanced field-emission behavior of layered MoS_2_ sheets. Small 9, 2730–2734 (2013). 23427106 10.1002/smll.201300002

[b25] Los J. H. . Melting temperature of graphene. Phys. Rev. B 91, 045415 (2015).

[b26] Chen S. S. . Oxidation resistance of graphene-coated Cu and Cu/Ni alloy. ACS Nano 5, 1321–1327 (2011). 21275384 10.1021/nn103028d

[b27] Zhu F. . Heating graphene to incandescence and the measurement of its work function by the thermionic emission method. Nano Res. 7, 553–560 (2014).

[b28] Starodub E., Bartelt N. C. & McCarty K. F. Viable thermionic emission from graphene-covered metals. Appl. Phys. Lett. 100, 181604 (2012).

[b29] Ni Z. H. . Probing charged impurities in suspended graphene using Raman spectroscopy. ACS Nano 3, 569–574 (2009). 19256543 10.1021/nn900130g

[b30] Hao Y. F. . Probing layer number and stacking order of few-layer graphene by Raman spectroscopy. Small 6, 195–200 (2010). 19908274 10.1002/smll.200901173

[b31] Hsu S. H. . Nanodiamond vacuum field emission device with gate modulated triode characteristics. Appl. Phys. Lett. 102, 203105 (2013).

[b32] Wei X. L. . Phonon-assisted electron emission from individual carbon nanotubes. Nano Lett. 11, 734–739 (2011). 21175216 10.1021/nl103861p

[b33] Wei X. L. . Electric-field-direction dependent spatial distribution of electron emission along electrically biased carbon nanotubes. Phys. Rev. B 84, 195462 (2011).

[b34] Lee J.-H. . Wafer-scale growth of single-crystal monolayer graphene on reusable hydrogen-terminated germanium. Science 344, 286–289 (2014). 24700471 10.1126/science.1252268

[b35] de Jonge N. & Bonard J.-M. Carbon nanotube electron sources and applications. Philos. Trans. R. Soc. Lond. A 362, 2239–2266 (2004). 10.1098/rsta.2004.1438 15370480

[b36] Wei X. L., Bando Y. & Golberg D. Electron emission from individual graphene nanoribbons driven by internal electric field. ACS Nano 6, 705–711 (2012). 22117647 10.1021/nn204172w

[b37] Kim Y. D. . Bright visible light emission from graphene. Nat. Nanotechnol 10, 676–681 (2015). 26076467 10.1038/nnano.2015.118

