# Supplementary material for: Tunable graphene micro-emitters with fast temporal response and controllable electron emission
Source: Nat Commun. 2016 May 10;7:11513. doi: 10.1038/ncomms11513 (PMC4866330; doi:10.1038/ncomms11513)
Supplement: Supplementary Information — Supplementary Figures 1-11, Supplementary Discussion and Supplementary References [file ncomms11513-s1.pdf]

## Supplementary Figures

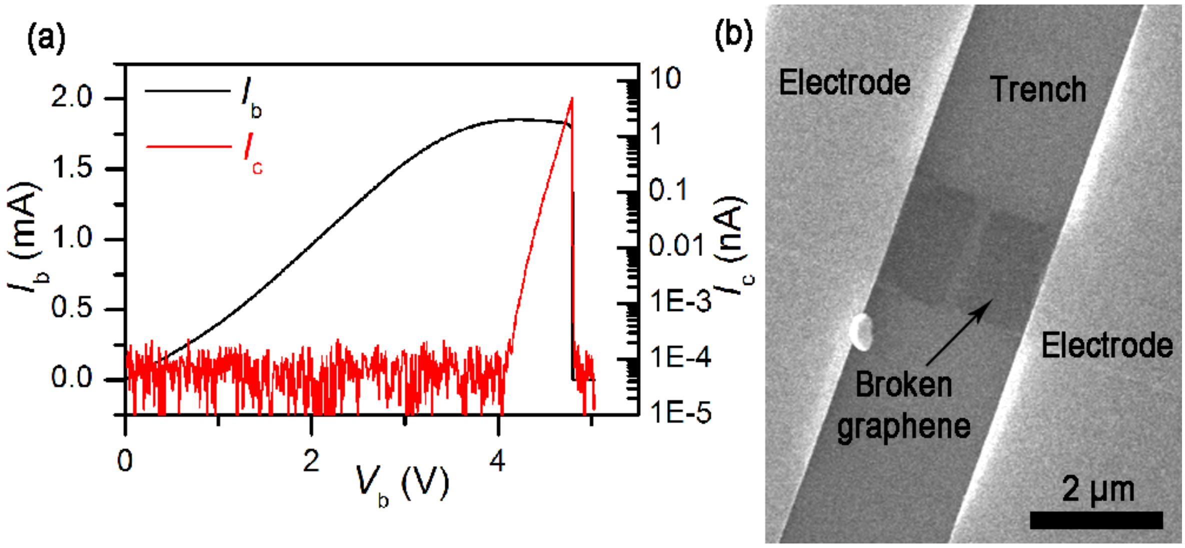


**Supplementary Figure 1.** (a) Simultaneously measured emission current ($I_c$) and current passing through graphene ($I_b$) of a graphene micro-emitter (GME) with $V_g$=0 V and $V_c$=100 V when ramping up $V_b$ until the breakdown of the GME at 4.80 V. (b) Scanning electron microscope (SEM) image of the GME taken after the measurement in (a) was completed, clearly showing that the graphene broke into two pieces. The synchronous dropping down of $I_c$ and $I_b$ in (a) provides a evident proof that our measured electron emission are really from graphene and that electron emission occurs only when a enough electric current/voltage is applied to graphene to heat it hot enough.

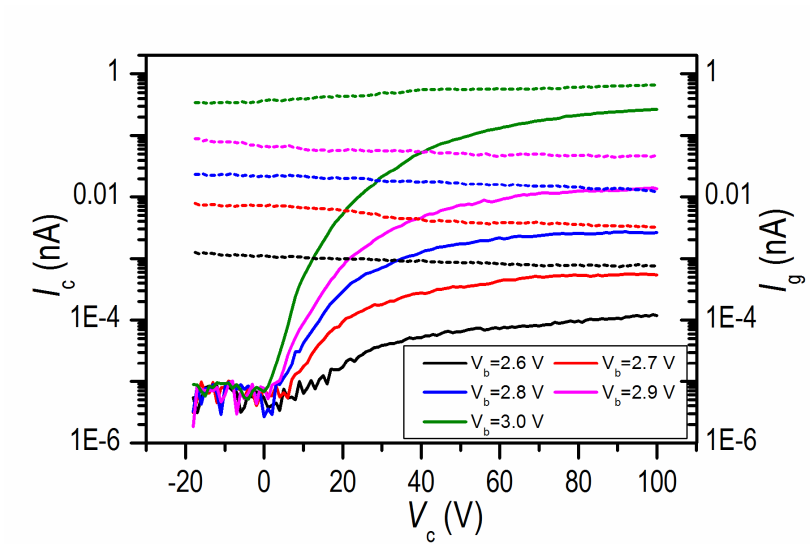


**Supplementary Figure 2.** The simultaneously measured $I_c$-$V_c$ (solid lines) and $I_g$-$V_c$ (dotted lines) curves of a GME when $V_b$ increased from 2.6 to 3.0 V at 0.1 V intervals and $V_g$ was fixed at 15 V. The $I_c$-$V_c$ curves exhibit typical emission features of a thermionic emitter under external electric field. When $V_c$ is negative, emission current measured by the top W electrode ($I_c$) is completely suppressed by a retarding electric field. When $V_c$ is larger than 0 V, $I_c$ becomes measurable and increase fast with the increase of $V_c$, exhibiting a space charge regime. When $V_c$ is further increased to approximately 30 V for the $I_c$-$V_c$ curve of $V_b$=2.6 V, it reaches the accelerating field regime where $I_c$ increases slowly with $V_c$ and approaches saturation. The threshold voltage for reaching accelerating field regime increases with $V_b$. At $V_b$=3.0 V, the $I_c$-$V_c$ curve reaches accelerating field regime at a $V_c$ of approximately 80 V. Therefore, a $V_c$ of 100 V is enough to ensure that all $I_c$-$V_g$ curves in Figure 1e were measured at accelerating field regime without the retardation of space charges.

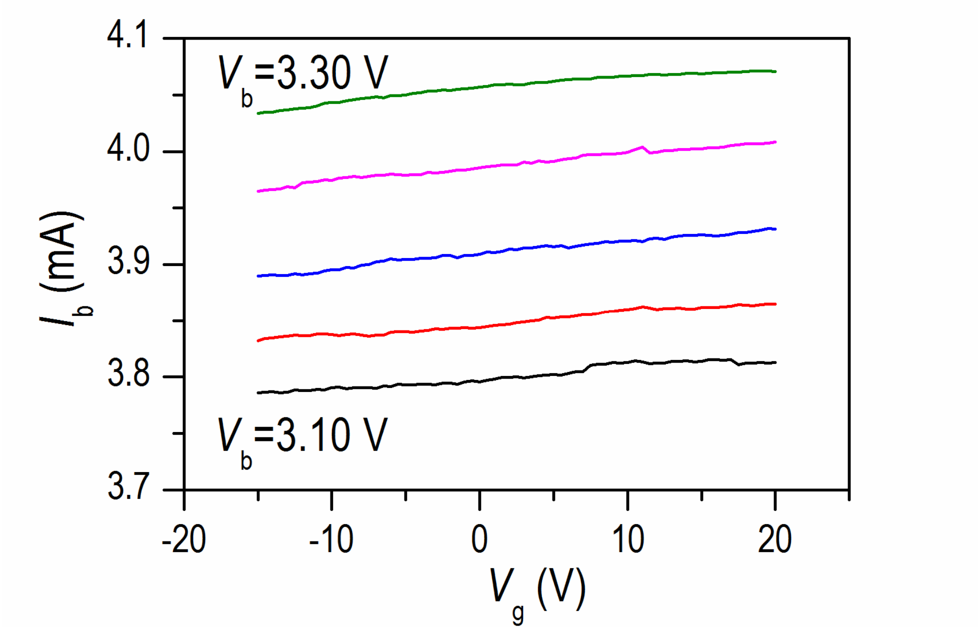


**Supplementary Figure 3.** $I_b$-$V_g$ curves of the same GME in Figure 1e when the applied bias voltage ($V_b$) increases from 3.10 to 3.30 V in 0.05 V intervals. It can be seen that the electrical conductivity or $I_b$ of the graphene exhibits weak tunability by $V_g$. So the large magnitude tunability of emission current up to 6 orders in Figure 1e cannot be attributed to the tunability of graphene conductivity.

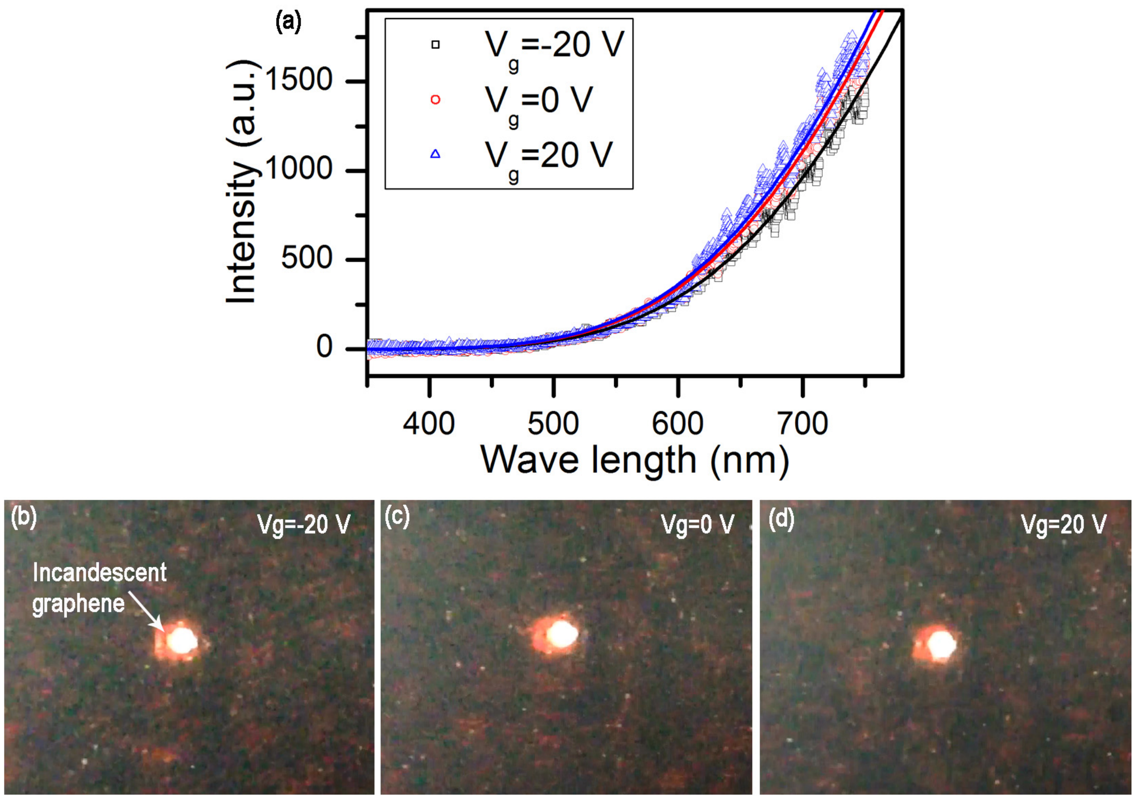


**Supplementary Figure 4.** (a) Thermal light emission spectra (symbols) of a GME when gate voltage is -20 V, 0 V and 20 V and the GME is driven by a bias voltage of 4.25 V.[1] Solid lines are the fitting of the spectra with Planck's law. By fitting the spectra with the Planck's law for black body irradiation,[1] we get the temperature of graphene as 1747 K, 1768 K and 1775 K, respectively, when gate voltage is -20 V, 0 V and 20 V. So the temperature of a graphene emitter under a fixed bias voltage exhibits a minor increase with the increase of gate voltage. When electron emission from graphene occurred, bright incandescent light emission from graphene could be observed. (b-d) Optical microscope images of an incandescent graphene when gate voltage is -20 V (b), 0 V (c) and 20 V (d), respectively. The magnification of those images is 17. The intensity of incandescent light emission from graphene was found to exhibit no obvious dependence on gate voltage.

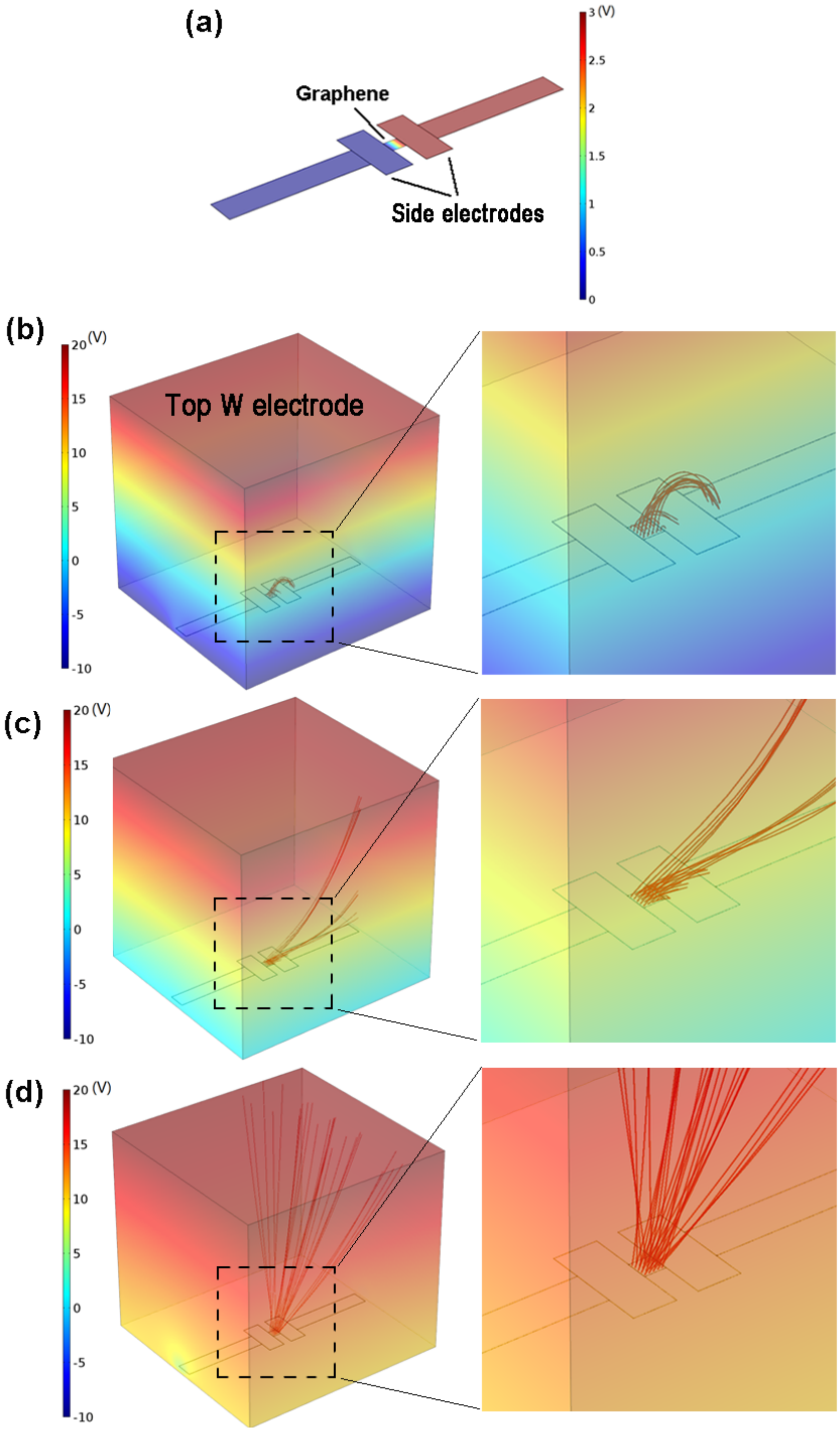


**Supplementary Figure 5.** Electron trajectories of a GME are simulated using COMSOL Multiphysics software. The simulation is performed in a vacuum space with a volume of 40 μm × 40 μm × 40.3 μm. The potential of the upper boundary of the simulated space is set to be $V_c$=20 V to simulate the top W electrode (the potential is set to make collecting electric field

(approximately 0.5 V μm$^{-1}$) be similar to that in our experiments). The potential of the bottom boundary of the simulated space is set to be $V_g$ to simulate the bottom gate. A graphene film with a dimension of 2 μm×1.8 μm × 3.4 nm and the two metal electrodes with a thickness of 70 nm are located at 0.3 μm above the bottom boundary in a symmetrical configuration with the graphene film being suspended between the two metal electrodes. One metal electrode is set to be grounded while the other one is set to be $V_b$=3.0 V. As shown in (a), a linear distribution of electric potential along graphene film is assumed with the potential at its two ends equal to that of the metal electrode contacting with it. Simulated electron trajectories in 3D space when $V_g$=-10 V, 1 V and 10 V, corresponding to the three regimes of $I_c$-$V_g$ curves in Figure 1e, are shown in (b), (c) and (d), respectively. The potential of simulated space is shown in color scale. An initial electron velocity of zero is assumed when simulating their trajectories. Electron trajectories are indicated by red solid lines. It can be seen that, when $V_g$=-10 V (b), electrons emitted from the grapheme film are all collected by the side metal electrode with a positive $V_b$ applied, and thus emission current measured by the top W electrode ($I_c$) is completely suppressed. When $V_g$=1 V (c), some electrons start to be projected to the top W electrode (top surface of the simulated space). At $V_g$=10 V (d), all electrons emitted from graphene film are projected to the top W electrode, so emission current measured by the top W electrode approaches saturation.

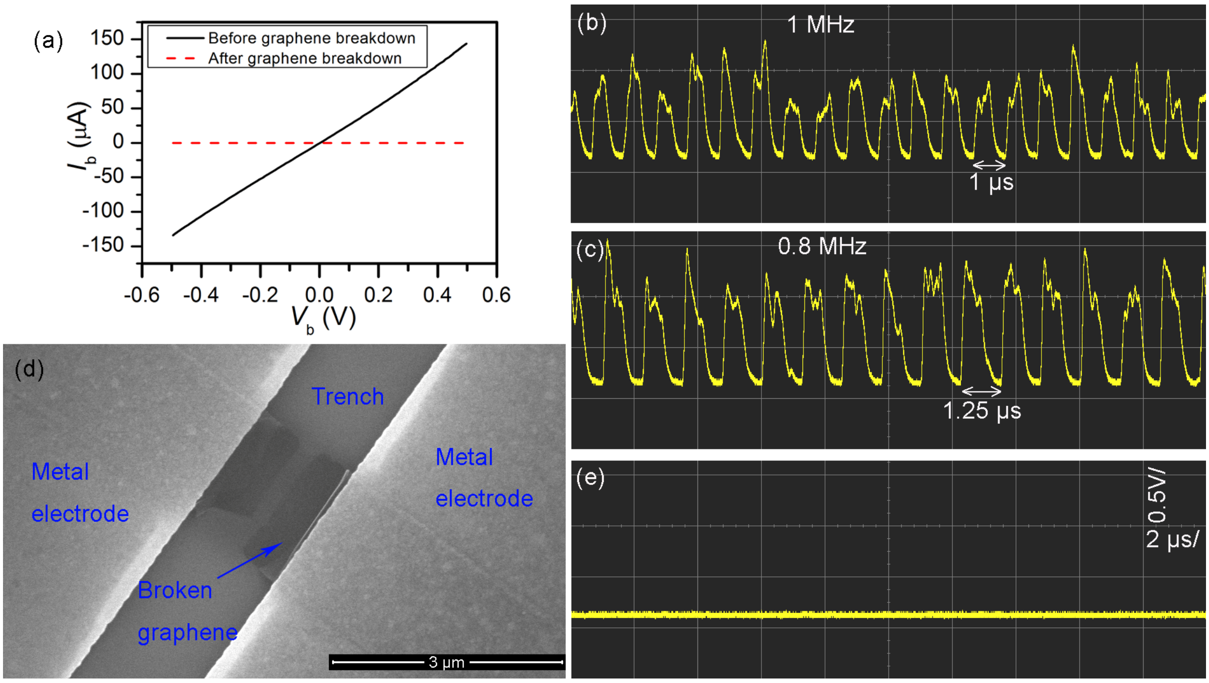


**Supplementary Figure 6.** (a) $I_b$-$V_b$ curves of a GME before and after the breakdown of the graphene film by extreme electric current stress. (b-c) The recorded ETD signals when a square wave voltage with a frequency of 1 MHz (b) and 0.8 MHz (c) was applied to the GME to enable its electron emission before its breakdown. The high and low voltage levels of square wave were set to be 2.0 V and 2.4 V, respectively. It can be seen that the frequency of the signal recorded by ETD are exactly same as that of input square wave voltage. (d) SEM image of the GME after the breakdown of the graphene film. (e) The output of ETD with all measurement parameter settings kept same as those in (c) after graphene film was broken down. The periodic signal as shown in (c) was not detected after the breakdown of graphene film. The comparative measurement results before (c) and after (e) the breakdown of the graphene film provide solid evidence that the signal of ETD as shown in (b) and (c) originates from electron emission from the GME but not an electric signal noise.

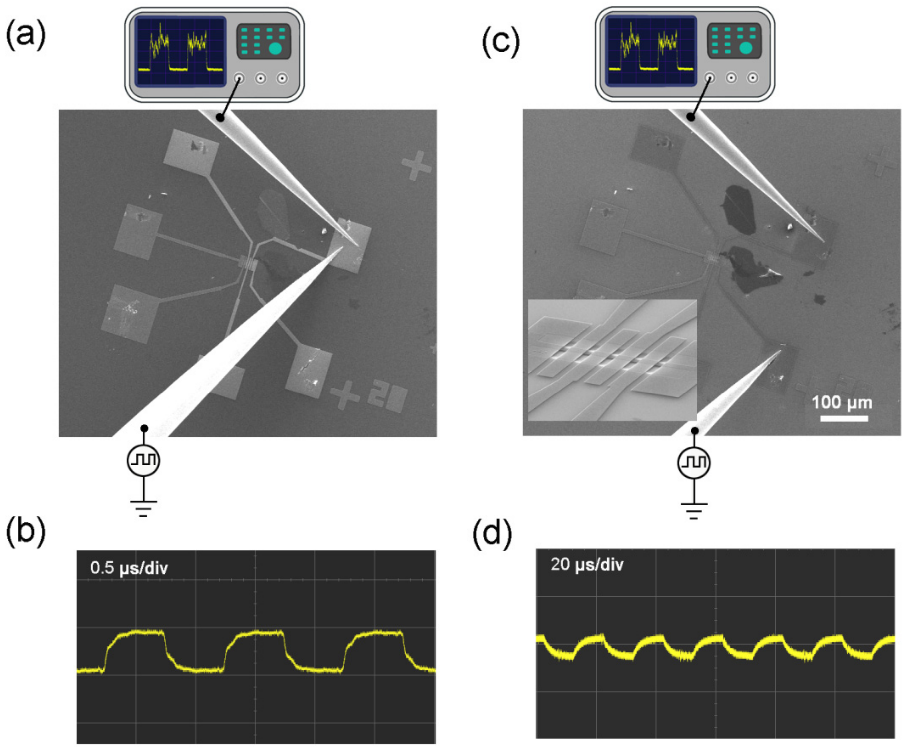


**Supplementary Figure 7.** The temporal response of a GME is measured by inputting a square wave signal to one Au/Ti electrode of the GME from a waveform generator (Agilent 33220A) through a nanoprobe system (Kleindiek MM3A) as shown in Figure 3a. So the input circuit of square wave voltage includes the feedthrough circuit of the nanoprobe system, the cable between the nanoprobe system and the waveform generator, and the microfabricated Au/Ti electrodes of a GME. We evaluate the temporal response of the input circuit without the microfabricated electrodes of a GME as shown in (a), where the two probes (one is connected to the waveform generator for signal input, the other one is connected to an oscilloscope for signal recording) connect directly to each other via a large metal pad. The recorded signal by an oscilloscope is shown in (b). It can be seen that the input circuit without the microfabricated electrodes has a response time of about 0.2 μs, close to that measured in Figure 3b in the main text. Then temporal response of the input circuit including the microfabricated electrodes of a GME is evaluated by making an input square-wave signal transmitted through a graphene micro-emitter as well as the external circuit as shown in (c). The response time in this case is shown in (d) and increases remarkably to about 10 μs. Therefore, our measurement system has a large impedance

and slow temporal response and seriously degrades our measured temporal response of GMEs. The determined temporal response of a GME as shown in Figure 3b in the main text is expected to be much slower than its intrinsic response. The inset in (c) is the enlarged SEM image of the centre part of the device, which consists of five GMEs.

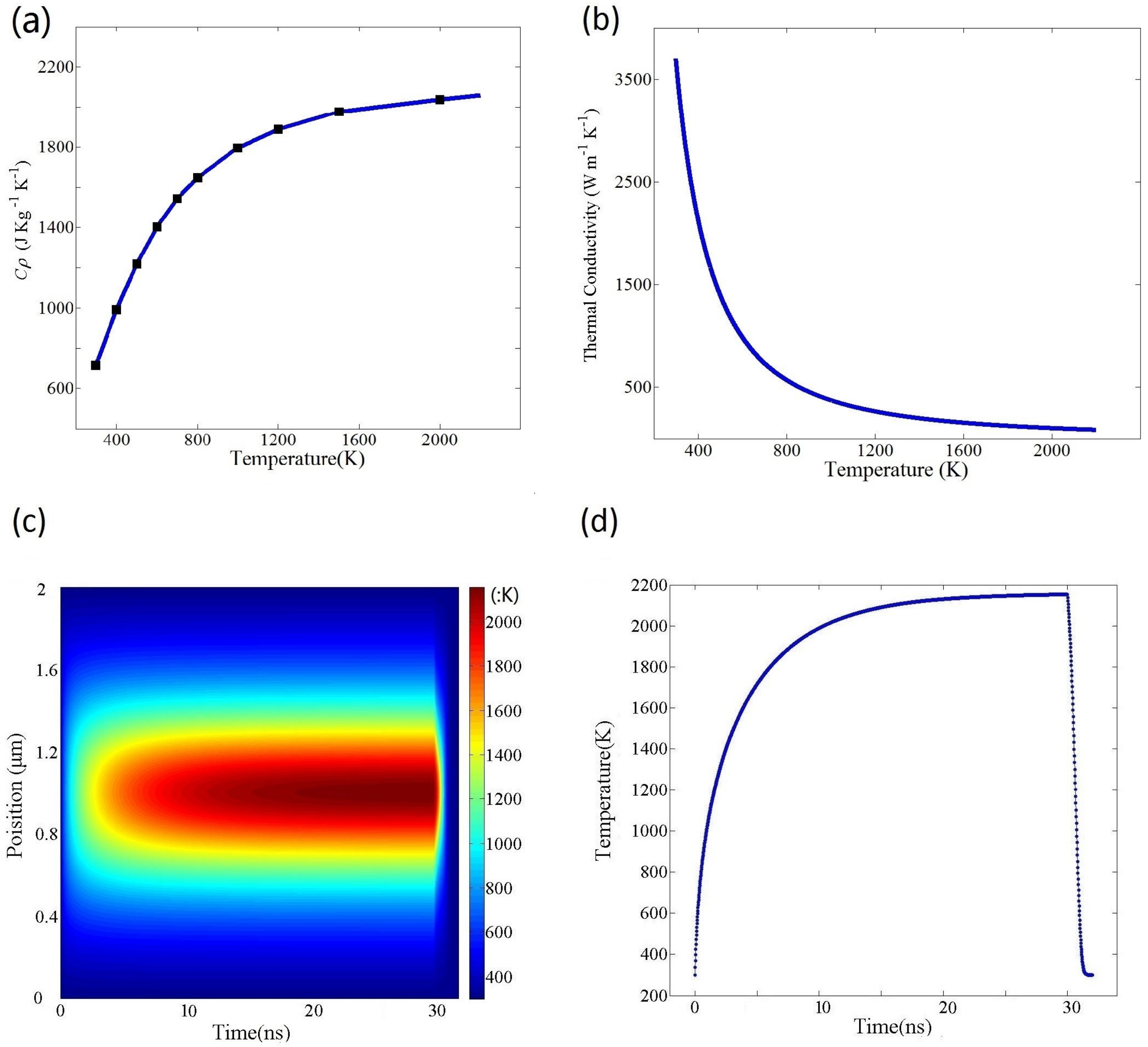


**Supplementary Figure 8.** (a) The specific heat of graphite used in temperature calculation. The dots are the reported value in Supplementary Reference 2, while the solid line is a fitted curve. (b) Thermal conductivity of exfoliated graphene used in the calculation.[3] **(**c) The calculated temperature distribution along a graphene film of 2.0 μm×2.0 μm and its evolution with time, under the Joule-heating of 3 V bias voltage and 0.93 mA electric current for a duration of 30 ns and the subsequent cooling. (d) Temperature evolution at the midpoint of the graphene film in (c). It can be seen that the graphene film can reach a steady temperature of approximately 2150 K in a time of 20 ns under Joule-heating and can be cooled down to room temperature in a time of only 1.3 ns.

The temperature distribution and evolution in Supplementary Figure 8 is calculated using following method. Assuming a GME has uniform temperature distribution along its width direction, its heating and cooling dynamics can be analyzed by using the thermal conduction equation in one-dimensional form:

$$C_\rho(\mathrm{T})\,\rho_d \frac{\partial T(x,t)}{\partial t} = \frac{\partial}{\partial x}\left(\kappa \frac{\partial T(x,t)}{\partial x}\right) - \frac{2(\mathrm{H}+W)\varepsilon\sigma(T(x,t)^4 - T_0^4)}{WH} + \frac{IV}{LWH}$$

Here, $C_\rho(\mathrm{T})$ is the volume specific heat capacity shown in Supplementary Figure 8a (we here use the value of graphite for it[2]). $\rho_d$=1060 kg $m^{-3}$ is the mass density of graphene.[4] $\kappa$ is the thermal conductivity at temperature $T$ and is described as $\kappa = \kappa_0 (T_0 / T)^{1.9}$ above the room temperature for exfoliated graphene with $\kappa_0$ =3700 $Wm^{-1}K^{-1}$ being the thermal conductivity at room temperature of $T_0$=300 K (Supplementary Figure 8b).[3] σ is the Stefan-Boltzmann constant. $\varepsilon$=0.023 is the black body radiation coefficient of graphene.[5] $I$, $V$, $L$, $W$ and $H$ are the electric current, bias voltage, length, width and thickness of graphene film. $x$ and $t$ are position coordinate and time, respectively. Assuming Joule-heating starts at $t$=0, we have the initial condition of $T(x,0)=T_0$ for Joule-heating. For cooling, we have initial condition of $T(x,t1)=T_s$ with $t1$ being the time of turning GME off ($V$=0) and $T_s$ being the steady temperature under Joule-heating. The two ends of graphene film contact with metal electrodes and are assumed to be fixed at room temperature, so we have the boundary condition of $T(0,t)=T(L,t)=T_0$. With the initial and boundary conditions, the temperature distribution and evolution of a graphene film under Joule-heating and cooling can be obtained by solving the thermal conduction equation.

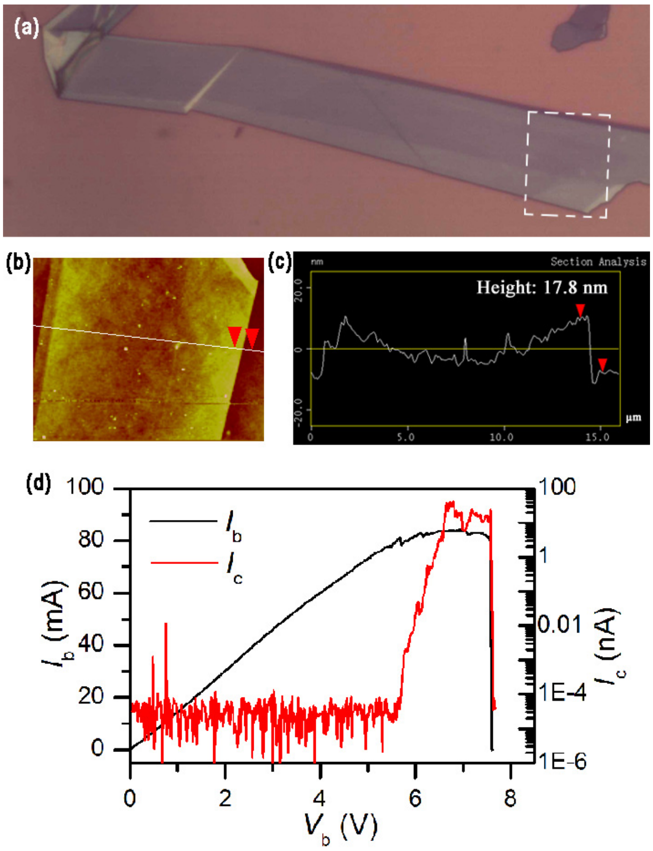


**Supplementary Figure 9.** (a) Optical microscope image of a thick graphene flake. (b) Atomic force microscope image of the framed area in (a). (c) Height profile of the graphene flake along the while line in (b), showing a thickness of 17.8 nm. This thickness corresponds to a layer number of approximately 52. (d) Electron emission performances of a GME fabricated from the graphene flake in (a) when $V_g$=15 V and $V_c$=100 V. The GME broke down at a $V_g$ of 7.6 V. It can be seen from the $I_c$-$V_b$ curve that electron emission from the graphene film occurs when $V_b$ is larger than approximately 5.5 V and a maximum emission current of 44 nA, corresponding to an emission density of 0.18 A cm$^{-2}$, is obtained. Therefore, the maximum emission current density of thick graphene films has similar magnitude to that of 1-2 layer graphene films. The maximum emission current density of graphene emitters was observed to exhibit no obvious dependence on their thickness. Furthermore, the $I_c$-$V_b$ curve in (d) exhibits a platform with remarkable fluctuation before breakdown. This platform may be caused by the layer-by-layer breakdown of graphene film. Since the surface area of thick graphene film keeps almost unchanged during the layer-by-layer breakdown, its emission current is expected to be approximately unchanged as well and thus exhibits a platform in the $I_c$-$V_b$ curve.

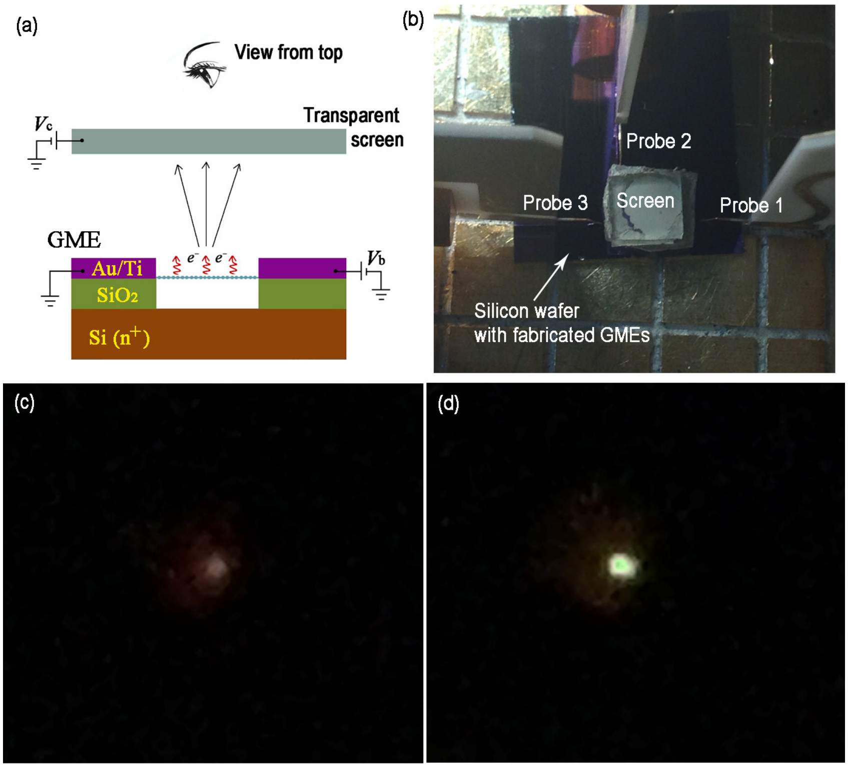


**Supplementary Figure 10.** (a) A side view schematic diagram of the measurement setup. A bias voltage ($V_b$) is applied to a GME to heat it up and enable electron emission. A transparent and electrical conducting fluorescent screen with a collecting voltage ($V_c$) applied is placed ~1 mm above the GME to collect electrons and display the graph of electron emission. (b) A top-view photograph of the measurement setup. The measurement was performed in a Lakeshore TTP4 probe station and three probes were used to achieve electrical connections. (c-d) The spot displayed on the fluorescent screen with no $V_c$ applied (c) and with a $V_c$ of 210 V applied (d). A bias voltage of 4.6 V was applied to the GME to enable its electron emission. When fluorescent screen was electrically floated with no $V_c$ applied, we observed a yellow pattern appearing on the fluorescent screen (c). This yellow pattern is attributed to incandescent light emission from hot graphene filament, since few electrons impinge on fluorescent screen in this case. When a $V_c$ of 210 V is applied to the fluorescent screen, electrons are accelerated and impinge on the fluorescent screen. A bright green spot was then observed on the fluorescent screen in addition to the yellow pattern due to incandescent light emission (d). The bright green spot is attributed to fluorescent light emission due to the impinge of energetic electrons on the fluorescent screen.

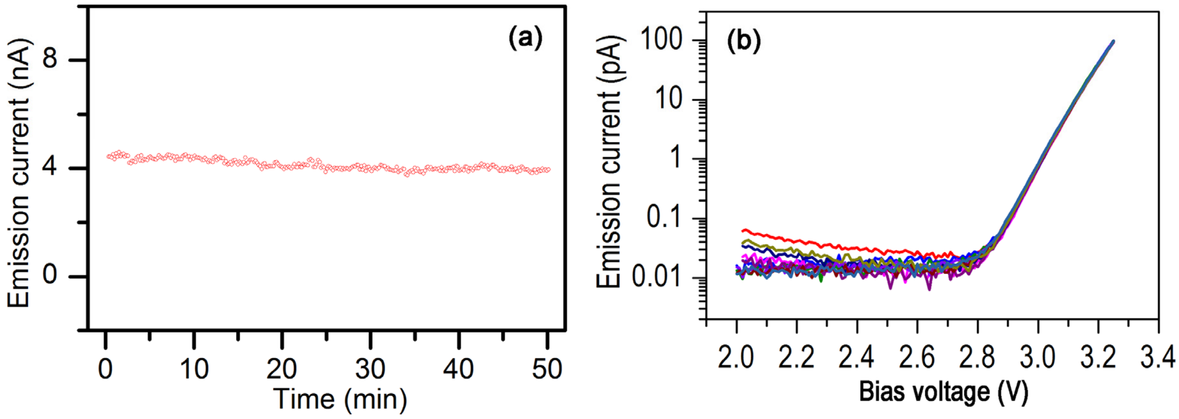


**Supplementary Figure 11.** (a) Emission current of a GME recorded in 50 minutes when bias voltage and collecting voltage are fixed. The GME have an emission current drift of 9% from 4.4 nA to 4.0 nA in 50 minutes, exhibiting good long-term emission stability in the relatively poor vacuum ($10^{-3}$ Pa). (b) Emission performances of a GME repeatedly measured by 11 times. It can be seen that the emission performances exhibit good stability with all the curves having the same turn-on voltage of approximately 2.8 V and exactly the same increasing rate of emission current with bias voltage. The measurements were performed at a vacuum level of approximately $10^{-3}$ Pa.

## Supplementary Discussion

We estimate the gate tunability of the graphene work function due to electrostatic doping by considering a monolayer graphene, which has a linear dispersion near the Dirac point. The relationship between the change of Fermi level and electron density can then be approximated to be $\Delta E_f \approx \sqrt{\pi \hbar^2 v_f^2 n}$ ($v_f$~ $1\times10^6$ m $s^{-1}$ is the Fermi velocity of graphene, $n$ is the electron density per unit area, and $\hbar$ is the reduced Planck constant).[6] The electron density per unit area under the tuning of gate voltage ($V_g$) can be written as $n = \frac{\varepsilon_0 V_g}{de}$ with $d$=300 nm being the distance between graphene emitter and the bottom gate, $\varepsilon_0$ being the vacuum permittivity, and $e$ being the elementary charge. For a $V_g$ of 20 V, $\Delta E_f$ is estimated to be 0.07 eV. This means that the Fermi level shifts upward by 0.07 eV relative to the energy band of graphene and also the vacuum level. Therefore, the work function of graphene is decreased by the same amount of 0.07 eV when $V_g$ is swept by 20 V. Assuming that emission current density is proportional to $\exp(-W/k_B T)$ with $W$ being work function, $k_B$ being the Boltzmann constant, and $T$ being the temperature of graphene measured to be ~1800 K (see Supplementary Figure 4a), the minor tuning of 30 K in graphene temperature (Supplementary Figure 4a) and 0.07 eV in work function just results in a tunability of emission current density by less than three times with a work function of 4.7 eV for graphene. Therefore, the large magnitude tunability of $I_c$ by $V_g$ as shown in Figure 1e cannot be attributed to the tunability of emission current density itself.

## Supplementary References:


1. Wei, X. L., Wang, S., Chen, Q. *et al.* Breakdown of Richardson's Law in Electron Emission from Individual Self-Joule-Heated Carbon Nanotubes. *Sci. Rep.* **4**, 5102-1-5 (2014).

2. Liu, P., Liu, L., Wei, Y. *et al.* Fast High-Temperature Response of Carbon Nanotube Film and Its Application as an Incandescent Display. *Adv. Mater.* **21**, 3563-3566 (2009).

3. Dorgan, V. E., Behnam, A., Conley, H. J. *et al.* High-field electrical and thermal transport in suspended graphene. *Nano Lett.* **13**, 4581-4586 (2013).

4. Rafiee M A, Rafiee J, Wang Z, *et al*. Enhanced mechanical properties of nanocomposites at low graphene content. *ACS Nano* **3**, 3884-3890 (2009).

5. Nair R R, Blake P, Grigorenko A N, *et al*. Fine structure constant defines visual transparency of graphene. *Science* **320**, 1308-1308 (2008).

6. Politano, A., Chiarello, G. Plasmon Modes in Graphene: Status and Prospect. *Nanoscale* **6**, 10927-10940 (2014).
